# Spatial features of specific CD103^+^CD8^+^ tissue-resident memory T cell subsets define the prognosis in patients with non-small cell lung cancer

**DOI:** 10.1186/s12967-023-04839-4

**Published:** 2024-01-05

**Authors:** Guanqun Yang, Siqi Cai, Mengyu Hu, Chaozhuo Li, Liying Yang, Wei Zhang, Jujie Sun, Fenghao Sun, Ligang Xing, Xiaorong Sun

**Affiliations:** 1 Shandong University Cancer Center, Shandong University, Jinan, Shandong China; 2 Department of Radiation Oncology, Shandong Cancer Hospital and Institute, Shandong First Medical University and Shandong Academy of Medical Sciences, Jinan, Shandong China; 3 Shandong Cancer Hospital and Institute, Shandong First Medical University and Shandong Academy of Medical Sciences, Jinan, Shandong China; 4 School of Clinical Medicine, Weifang Medical University, Weifang, Shandong China; 5 Department of Pathology, Shandong Hospital and Institute, Shandong First Medical University and Shandong Academy of Medical Science, Jinan, Shandong China; 6 Department of Nuclear Medicine, Shandong Cancer Hospital and Institute, Shandong First Medical University and Shandong Academy of Medical Sciences, Jinan, Shandong China

**Keywords:** CD103, Tissue-resident memory T cell, Tumor microenvironment, Multiplex immunofluorescence, Prognostic biomarker, NSCLC

## Abstract

**Background:**

Tissue-resident memory T (T_RM_) cells can reside in the tumor microenvironment and are considered the primary response cells to immunotherapy. Heterogeneity in functional status and spatial distribution may contribute to the controversial role of T_RM_ cells but we know little about it.

**Methods:**

Through multiplex immunofluorescence (mIF) (CD8, CD103, PD-1, Tim-3, GZMB, CK), the quantity and spatial location of T_RM_ cell subsets were recognized in the tissue from 274 patients with NSCLC after radical surgery. By integrating multiple machine learning methods, we constructed a T_RM_-based spatial immune signature (T_RM_-SIS) to predict the prognosis. Furthermore, we conducted a CD103-related gene set enrichment analysis (GSEA) and verified its finding by another mIF panel (CD8, CD103, CK, CD31, Hif-1α).

**Results:**

The density of T_RM_ cells was significantly correlated with the expression of PD-1, Tim-3 and GZMB. Four types of T_RM_ cell subsets was defined, including T_RM1_ (PD-1^−^Tim-3^−^T_RM_), T_RM2_ (PD-1^+^Tim-3^−^T_RM_), T_RM3_ (PD-1^−^Tim-3^+^T_RM_) and T_RM4_ (PD-1^+^Tim-3^+^T_RM_). The cytotoxicity of T_RM2_ was the strongest while that of T_RM4_ was the weakest. Compare with T_RM1_ and T_RM2_, T_RM3_ and T_RM4_ had better infiltration and stronger interaction with cancer cells. The T_RM_-SIS was an independent prognostic factor for disease-free survival [HR = 2.43, 95%CI (1.63–3.60), *P* < 0.001] and showed a better performance than the TNM staging system for recurrence prediction. Furthermore, by CD103-related GSEA and mIF validation, we found a negative association between tumor angiogenesis and infiltration of T_RM_ cells.

**Conclusions:**

These findings reveal a significant heterogeneity in the functional status and spatial distribution of T_RM_ cells, and support it as a biomarker for the prognosis of NSCLC patients. Regulating T_RM_ cells by targeting tumor angiogenesis may be a potential strategy to improve current immunotherapy.

**Supplementary Information:**

The online version contains supplementary material available at 10.1186/s12967-023-04839-4.

## Introduction

Despite advances in improvements in diagnostics and treatment, lung cancer remains the leading cause of cancer-related mortality worldwide, with approximately 61% (early-stage) ~ 34% (local-advanced) of 5-year survival rate, and non-small cell lung cancer (NSCLC) counts for over 80% of all lung cancer cases [1]. About 22% (stage I) ~ 68% (stage III) patients with operable NSCLC suffer from recurrence within 5 years, mainly in the first 2 years, indicating treatment failure in a considerable number of cases [2, 3]. The tumor microenvironment (TME), containing cancer cells, multifarious immune cells, vascular, mesenchymal cells and non-cell components, contributes greatly to the development of NSCLC. Consequently, there is an urgent need to improve the understanding of the TME of NSCLC for better patient stratification and recognizing novel targets to improve therapies.

Tissue-resident memory T cells (T_RM_) are a special population of immune cells that persistently reside in barrier tissues (skin, intestine and lung), lymphoid organs as well as solid tumors, patrol their microenvironment, and rapidly respond to alarm signals [4, 5]. T_RM_ cells are characterized by their expression of CD103 integrin, mediating the adhesion of intraepithelial T-cells to epithelial cells [6]. Emerging evidence has linked the quantity of T_RM_ cells, mostly CD103^+^CD8^+^T_RM_ (referred to by “T_RM_” in this study unless otherwise noted), to better response to immunotherapy and better prognosis of patients with lung cancer [7–9]. However, there are also different opinions that T_RM_ present a immunoregulatory characterization and exert a positive impact on tumor progression [10–12]. Of note, deep sequencing of T_RM_ in human lung cancer has revealed that T_RM_ appears to be functionally exhausted to varying degrees, expressing multiple immune checkpoints, especially PD-1 and Tim-3, which suggests that T_RM_ cells are highly heterogeneous and may be a potential target of immune checkpoint blockade [8, 13, 14]. Nonetheless, heterogeneity of T_RM_ functional subsets remains unknown.

Spatial architecture of the TME is increasingly considered extremely important to orchestrate tumor immunity and therapeutic response [15]. It has been recognized that CD8^+^ T cells in the invasive margin (IM) were more abundant but more dysfunctional than those in the tumor center (TC) [16, 17]. Likewise, a higher density of T cells and their different prognostic effects were observed in the stroma compartment compared to the epithelium compartment in NSCLC [18–20]. Additionally, intercellular interactions, as indicated by the spatial distance between cells, have received increasing attention. Some parameters, such as the nearest neighboring distance [21, 22] and proximity score (also known as an effective score or mixing score) [17, 23, 24], have been proposed to quantify this spatial interaction. Previous studies, including ours, have found that the poor spatial interaction between intratumoral CD8^+^T cells and cancer cells was correlated with lymph node metastases, recurrence and worse survival in patients with NSCLC [17, 25, 26]. Recently, approaches to comprehending the spatial architecture of the TME are transitioning from traditional single-color immunohistochemistry (IHC) to multiplex IHC/multiplex immunofluorescence (mIF), enabling the quantification and localization of multiple cell phenotypes simultaneously at single-cell resolution [27]. In this context, exploring the spatial architecture of T_RM_ functional subsets should not be absent.

Herein, we determined the density and spatial localization of CD103^+^CD8^+^ T_RM_ subsets with different immune checkpoint coexpression patterns through mIF in a cohort of patients with operable NSCLC, and revealed their significant heterogeneity.

## Materials and methods

### Patient cohort and sample collection

Formalin-fixed, paraffin-embedded (FFPE) tissue specimens and clinical information were retrospectively collected from NSCLC patients who underwent radical surgery at Shandong Cancer Hospital & Institute (SCHI, Shandong, China) between 2014 and 2018. Patients treated with preoperative antitumor therapy were excluded from this cohort. The final analysis included only lung adenocarcinoma (LUAD) and lung squamous cell carcinoma (LUSC) because of the small number of patients with other histological types (n = 5). This study had the approval of the Institutional Ethics Committee (SDTHEC2022007013).

### Tissue microarray construction and mIF staining

Tissue microarrays (TMA) were constructed from FFPE tissues as described in our previous studies [17, 26]. mIF staining was conducted with Akoya-Opal-Kit (Akoya Biosciences, U.S.A.; cat. NEL871001KT) following a previously described protocol [17, 26]. Briefly, 3 μm slides obtained from the TMA blocks were subjected to a deparaffinization using xylene and a rehydration through a graded ethanol series. Then, an antigen retrieval was performed using microwave incubation with citric acid buffer or EDTA buffer. Each slide underwent multiple rounds of staining, which involved antigen blocking with 1% BSA, reaction with specific primary antibodies and corresponding secondary antibodies, and covalent binding of tyramide signal amplification (TSA) fluorophores. Then antibody complexes were removed by the subsequent antigen retrieval before a new staining round. After all rounds of staining, the slides were counterstained with DAPI and scanned using the Vectra Polaris Scanner System (PerkinElmer, U.S.A.). Detailed information on the antibodies and reagents used can be found in Additional file 1: Table S1.

### Automatic image processing and immune-related feature definition

Multispectral images were processed in the Inform software 2.4.8 (PerkinElmer, U.S.A.) following a previously described procedure [17, 26]. In brief, after multispectral images unmix into single-spectral images, tissue segmentation into the epithelium compartment and stroma compartment, cell segmentation and phenotype identification, single-marker phenotypes and single-cell two-dimensional coordinates can be generated. Both the trained tissue segmentation classifier and the cell phenotype classifier had an accuracy of more than 98%. At least two investigators, including an experienced pathologist, independently reviewed all images.

To reveal the characteristics of T_RM_ cells, we calculated the number of all types of cell phenotypes of interest (Additional file 1: Table S2) and intercellular Euclidean distances. The cell density was determined by normalizing the corresponding cell count to the total cell counts (cell / 1000 cells) using the following formula:

$$Dens\, (A)=n\left(A\right)/n\left(total\, cells\right)\times 1000$$

where A represents one cell phenotype, and n corresponds to the number of cells. To assess the spatial characteristics of T_RM_, we established three distinct spatial features. The infiltration score (InS) was defined as the ratio of cell count between the epithelial and stromal compartment, reflecting the level of immune cell infiltration, whose formula was:

$$InS (A)=n{\left(A\right)}^{epi}/n{\left(A\right)}^{stro}$$

The mean nearest neighboring distance (mNND) from A cells to cancer cells was defined as the mean distance between each A cell and its nearest neighboring cancer cell, which was negatively related to the interplay intensity between them. The formula of mNND was:

$$mNND\left(A\sim C\right)=\sum_{i=n}{d}_{min}\left({A}_{i}\sim C\right)/n\left(A\right)$$

where C cell represents the cancer cell, and d_min_ (A_i_ ~ C) represents the minimum distance from the i-th A cell to its nearest neighboring cancer cell. The cancer-cell proximity score (CCPS) of A cells was defined as the average number of A cells within a predefined radius of all cancer cells. The formula of mNND was:

$$CCPS\left(A\right)=\sum_{i=N}n\left({C}_{i}\stackrel{r}{\to }A\right)/N\left(C\right)$$

in which r represents a predefined radius, and $n\left({C}_{i}\stackrel{r}{\to }A\right)$ represents the number of A cells within r-μm radius of the i-th cancer cell. A higher CCPS of A cells indicated more effective A cells interplayed with cancer cells.

### Prognostic signature construction

The entire cohort was randomly divided into a training set and a testing set at a ratio of 7: 3 according to the 10 events per variable (10 EPV) principle. Firstly, in the training set, we performed an importance evaluation of candidate immune-related features through multiple machine learning algorithms, including Least Absolute Shrinkage and Selection Operator (LASSO) regression algorithm (tenfold cross-validations), eXtreme Gradient Boosting (XGBoost) algorithm (1000-time repetitions) and Random Forest (RF) algorithm. The intersection of their respective top 10 important features was selected to construct a T_RM_-based spatial immune signature (T_RM_-SIS) in the Cox regression model:

$$SIS=\sum_{i=1}^{n}{\text{ln}}\left({HR}_{{X}_{i}}\right)\times {X}_{i}$$

where in ${HR}_{{X}_{i}}$ is the HR for prognostic feature X_i_ selected by the above procedure.

### Verification of partial results in a public cohort

To verify our results on CD103 coexpression with checkpoints and partially validate our prediction model, we constructed an external cohort by collecting samples from The Cancer Genome Atlas (TCGA) database based on the same inclusion criteria as the SCHI-NSCLC cohort. We followed the principles of the TCGA Research Network to process the data and retained only those cases with complete mRNA expression information and clinical prognostic information [28].

### Gene Set Enrichment Analysis (GSEA)

To determine the CD103-related biological pathways, we collected a suitable Gene Expression Omnibus (GEO) dataset (GSE10245) and conducted GSEA in official GSEA software (V.4.3.2) with default parameters. Samples of the GEO dataset were divided into two groups evenly based on the median standardized counts of CD103 mRNA expression. Gene sets in “C5: GOBP” collection from the Human Molecular Signatures Database (MSigDB) were applied. A gene set would be considered statistically significant if the absolute value of its normalized enrichment score (NES) was over 1.00 and its false discovery rate (FDR) q-value was below 0.05.

### Statistical Analyses

The Mann–Whitney U tests for two independent samples and Kruskal–Wallis tests for multiple samples were used to analyze the differences in immune-related features and clinicopathological characteristics. Spearman rank correlation coefficients were used to evaluate the correlation among these variables. Disease-free survival (DFS) was defined as the time from the first day of radical surgery to a progression event or last follow-up. To evaluate the predictive performance of the T_RM_-SIS, the areas under the curve (AUCs) of time-dependent receiver operating characteristic (ROC) curves were applied. The survival analyses were conducted by Kaplan–Meier plot and log-rank test between patients with high T_RM_-SIS and low T_RM_-SIS. We used the univariate and multivariate Cox proportional hazard regression models (backward elimination) to estimate the independent prognostic value of the T_RM_-SIS. A two-sided *P* value less than 0.05 was considered statistically significant. All statistical results were reviewed by at least two investigators independently. All statistical analyses were carried out in R 4.2.3 and SPSS 26.0 (IBM, U.S.A.).

## Results

### Demographic characteristics of the NSCLC cohort

A total of 274 patients with stage IA-IIIB NSCLC meeting the inclusion criteria were eventually enrolled, among which 108 cases (39.4%) relapsed during the follow-up. The median follow-up time was 65.9 months. The majority of the cases were male (65.0%), with stage I disease (55.3%) and LUAD (65.3%) (Additional file 1: Table S3).

### Quality control evaluation of mIF

To ensure that the mIF project we developed was effective and stable, we conducted a quality control (QC) evaluation through a QC tool from the manufacturer (Akoya Biosciences, U.S.A.). It demonstrated an acceptable heterogeneity of signal intensity across multispectral images, low-level crosstalk among spectral channels and accurate unmixing from noise, indicating a good performance of the mIF project (Additional file 1: Figure S1, S2, Additional file 1: Table S4).

### TRM cells possess better infiltration and stronger interaction with cancer cells than T_non-RM_ cells

**Fig. 1:**
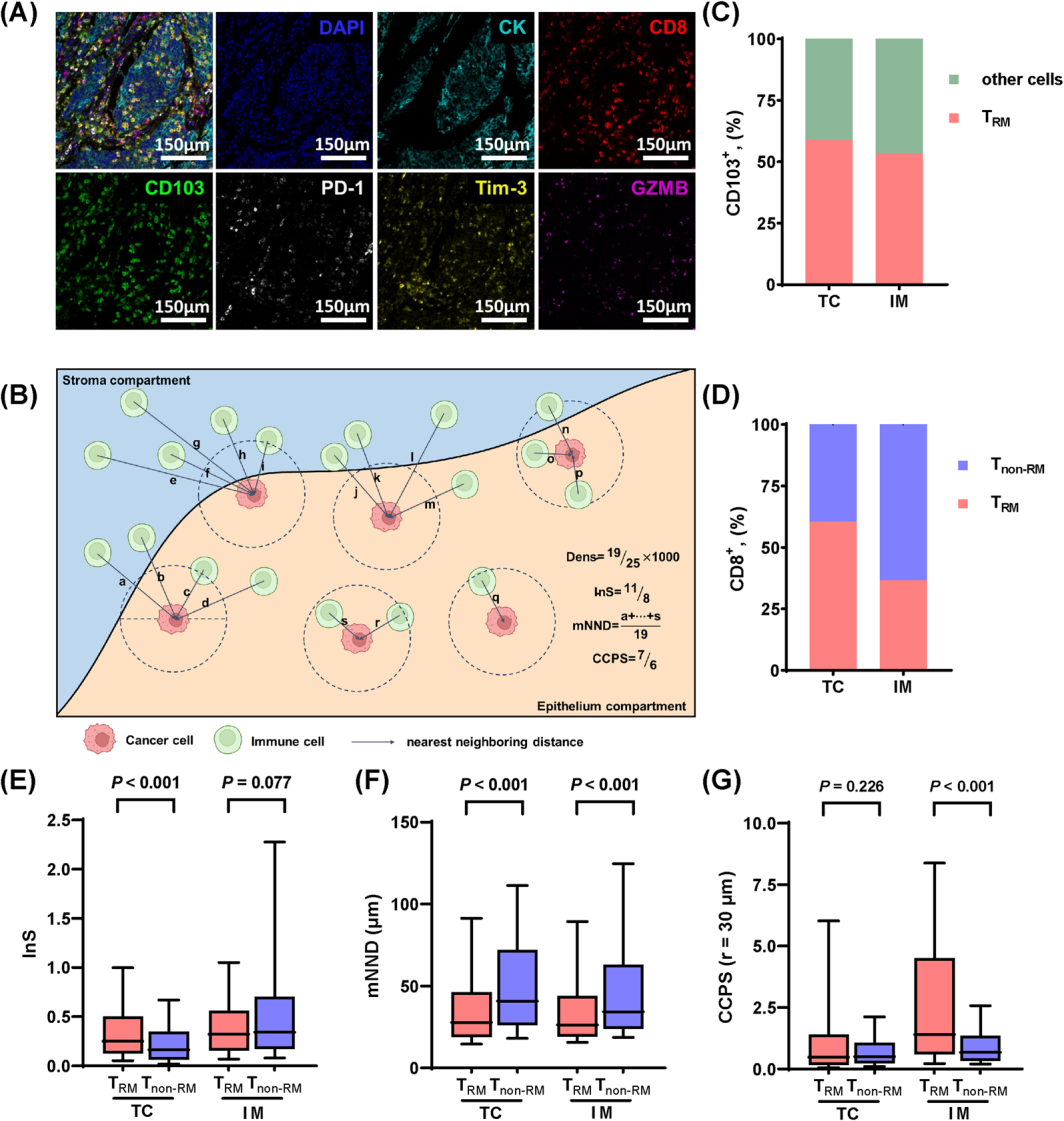
Multiple immunofluorescence (mIF) identifies T_RM_ and their spatial infiltration features in patients with NSCLC. **A** The merged and single-stained images for the panel of mIF. **B** Diagram illustrating the calculation of cell density (Dens), infiltration score (InS), mean nearest neighboring distance (mNND) and cancer-cell proximity score (CCPS). Line a ~ s represent the nearest distance from each immune cell to its neighboring cancer cell when calculating mNND. **C** Proportion of CD8^+^CD103^+^ cells (T_RM_ cells) in total CD103^+^ cells. **D** Proportion of CD8^+^CD103^+^ cells (T_RM_ cells) T_RM_ in total CD8^+^ cells. **E–G** Differences of spatial infiltration features between T_RM_ and T_non-RM_. Statistical significance was calculated using the Mann–Whitney U test

Based on previous results from single-cell sequencing [8, 13, 14], we employed mIF to identify cytotoxic T cells (CD8^+^ cells), T_RM_ cells (CD103^+^CD8^+^ cells), T_non-RM_ cells (CD8^+^CD103^−^ cells), immune checkpoints (PD-1, Tim-3), cytotoxic indicator (GZMB) and cancer cells (CK^+^ cells) (Fig. 1A). As mentioned earlier, three spatial parameters were defined to describe the spatial distribution of cells (Fig. 1B, C). T_RM_ accounted for more than half of CD103^+^ cells both in TC and in IM (58.87% and 53.21%, respectively), indicating that cytotoxic T cells were the primary source of CD103 expression (Fig. 1C). T_RM_ cells predominated cytotoxic T cells in TC (60.50%) but vice versa in IM (36.55%) (Fig. 1D). Compared with T_non-RM_, T_RM_ cells possessed higher InS in TC (*P* < 0.001), shorter mNND to cancer cells in both TC (*P* < 0.001) and IM (*P* < 0.001), and higher CCPS in IM (*P* < 0.001), reflecting better infiltration and stronger interaction with cancer cells of T_RM_ (Fig. 1E–G).

### TRM subsets defined by immune checkpoint coexpression patterns presented discrepant functional states and spatial architecture

**Fig. 2:**
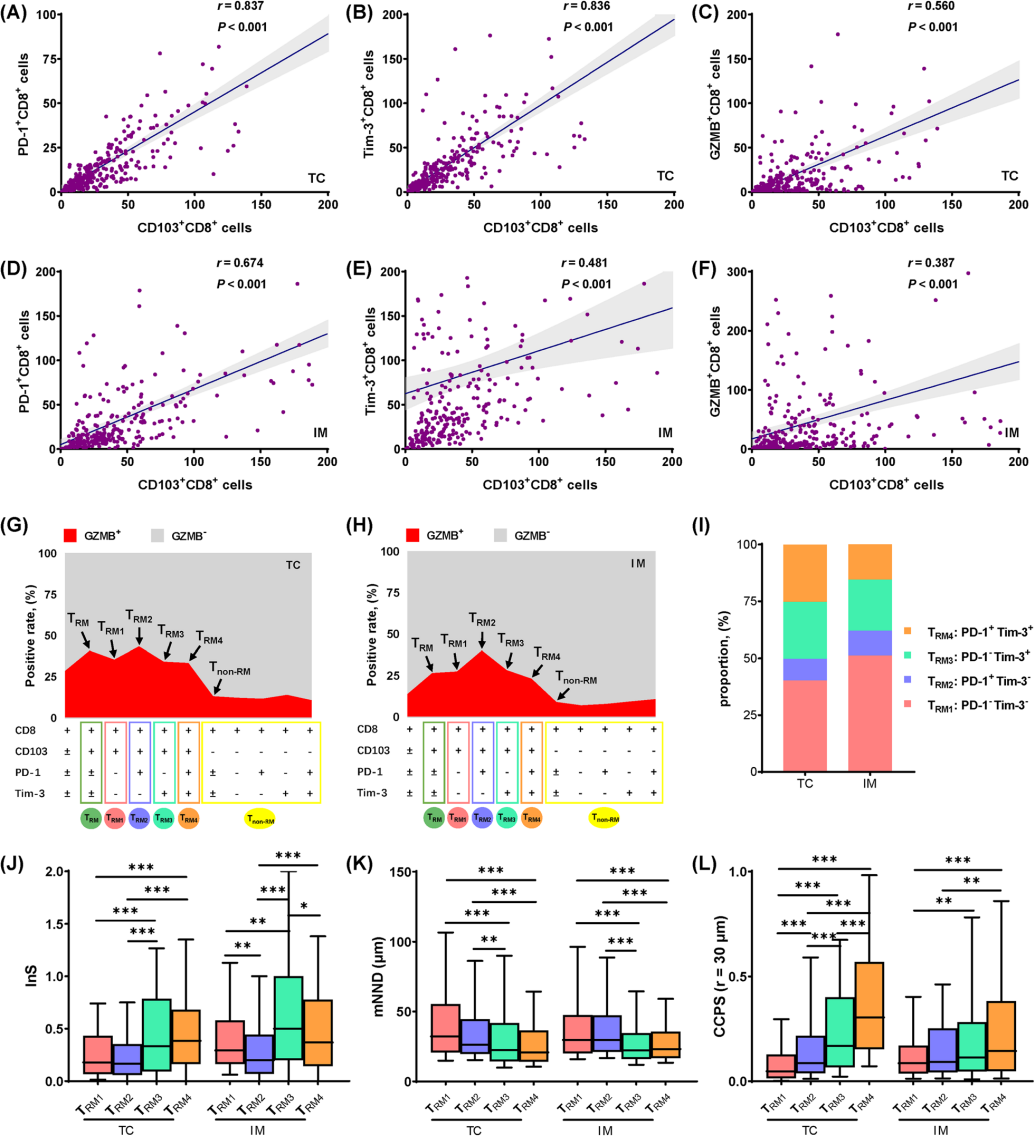
Definition of different T_RM_ cell subsets in patients with NSCLC. **A**–**F **Close positive correlation of CD103 and immune-related molecules (PD-1, Tim-3 and GZMB) on CD8^+^T cells. The correlation was calculated using the Spearman rank correlation coefficient. **G**–**H** Discrepant cytotoxic abilities among different T_RM_ cell subsets defined by different immune checkpoint coexpression patterns. **I** Composition of four types of T_RM_ subsets. **J**–**L** Differences of spatial features among T_RM_ subsets. Statistical significance was calculated using the Kruskal–Wallis test. *P < 0.05; **P < 0.01; ***P < 0.001

Close interaction between T cells and cancer cells can induce the exhaustion of T cells [29]. The expression of immune checkpoints could reflect the functional state of T cells [14]. We found that CD103^+^CD8^+^ cells showed significant positive correlations with PD-1^+^CD8^+^ cells, Tim-3^+^CD8^+^ cells and GZMB^+^CD8^+^ cells (Fig. 2A–F). Considering the specific phenotype of T_RM_ in GZMB production, we compared the cytotoxic capacity among different CD8^+^T cell subsets by the proportion of GZMB^+^ cells [5, 30]. The proportion of GZMB^+^ cells reached its highest in PD-1^+^Tim-3^−^T_RM_ and lowest in PD-1^+^Tim-3^+^T_RM_, where it was still much higher than that in T_non-RM_ subsets, indicating a considerable heterogeneity of cytotoxicity among CD8^+^ T-cell subsets with different immune checkpoint expression patterns (Fig. 2G, H). Consequently, based on the different immune checkpoint expression patterns, we divide T_RM_ into four subgroups: PD-1^−^Tim-3^−^T_RM_ (T_RM1_), PD-1^+^Tim-3^−^T_RM_ (T_RM2_), PD-1^−^Tim-3^+^T_RM_ (T_RM3_), and PD-1^+^Tim-3^+^T_RM_ (T_RM4_). In TC, four subsets accounted for 40.21%, 9.52%, 25.04% and 25.23% of T_RM_, respectively. In IM, there was more T_RM1_ (51.23%) and less T_RM4_ (15.45%) than TC (Fig. 2I). Afterward, we compared the spatial features in pairs among four T_RM_ subsets. The InS of T_RM1_ and T_RM2_ were significantly lower than those of T_RM3_ and T_RM4_. Notably, the mNND to cancer cells progressively decreased from T_RM1_ to T_RM4_ whereas the CCPS progressively increased from T_RM1_ to T_RM4_ (Fig. 2J–L). These findings evidenced spatial and functional heterogeneity in T_RM_ cell subsets.

### Construction of TRM-based spatial immune signature by integration of multiple machine learning methods

**Fig. 3:**
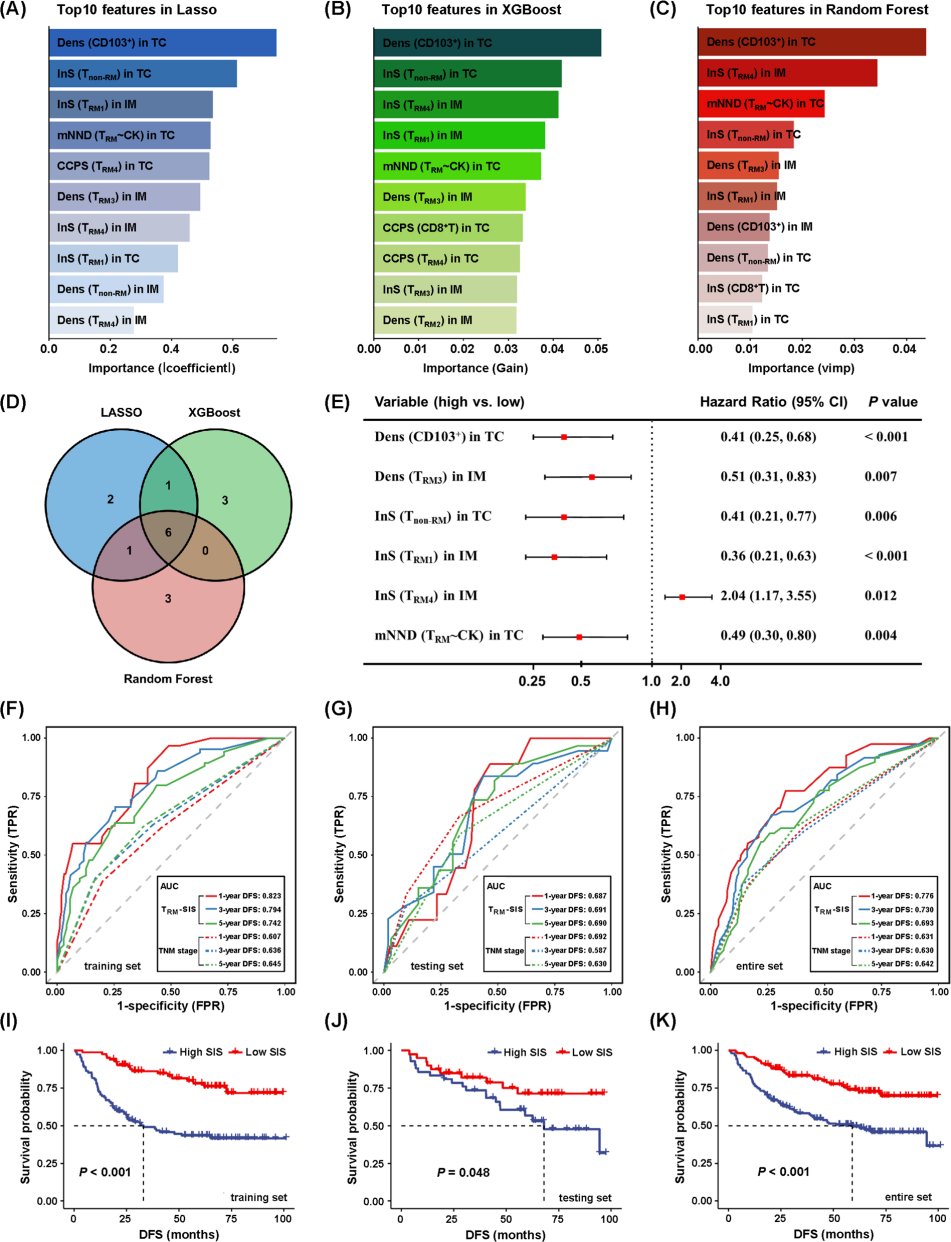
Construction of immune scoring system by integrating multiple machine learning methods. **A**–**C** The importance feature maps show the top 10 important features in LASSO model, XGBoost model and Random Forest model. **D** Venn diagram demonstrates the six immune features shared by the three models: density of CD103^+^ cells in TC, density of T_RM3_ in IM, infiltration score of T_non-RM_ in TC, infiltration score of T_RM1_ in IM, infiltration score of T_RM4_ in IM and mean nearest neighboring distance from T_RM_ to cancer cells in TC. **E** Univariate Cox analyses show the prognostic relevance of the six selected features. **F**–**H** Time-dependent ROC curves and AUC values of T_RM_-SIS system and TNM staging system for prediction of recurrence risk at 1, 3 and 5 years in the training set, testing set and entire cohort. The T_RM_-SIS system presents better performance than the TNM staging system for recurrence prediction. **I**–**K** Survival analyses based on the T_RM_-SIS in the training set, testing set and entire cohort. Statistical significance was calculated using the log-rank test. AUC, area under the curve; DFS, disease-free survival; ROC, receiver operating characteristic

The entire cohort (n = 274) was randomly divided into a training set (n = 192) and testing set (n = 82), and no clinicopathological factors were significantly different between them (Additional file 1: Table S5; Additional file 2: Suppl_data1). For a comprehensive assessment of T_RM_, we constructed the T_RM_-SIS via integrating three machine learning algorithms, including LASSO, XGBoost and Random Forest. We firstly assessed and ranked the importance of all candidate T_RM_-related features by the three algorithms, respectively (Fig. 3A–C). In order to select the robust features, we took the intersection features of the three sets of top 10 important features as the variables for constructing the T_RM_-SIS and finally screened out six important features: density of CD103^+^ cells in TC, density of T_RM3_ in IM, InS of T_non-RM_ in TC, InS of T_RM1_ in IM, InS of T_RM4_ in IM and mNND from T_RM_ to cancer cells in TC (Fig. 3D, E). Their cut-off values were determined using X-tile software (Yale University, U.S.A.) [31]. The T_RM_-SIS was calculated for each patient according to the linear combination of these features weighted by their coefficients of the univariate Cox analyses: T_RM_-SIS = [ln0.41 × Dens(CD103) in TC] + [ln0.51 × Dens(T_RM3_) in IM] + [ln0.41 × InS(T_non-RM_) in TC] + [ln0.36 × InS(T_RM1_) in IM] + [ln2.04 × InS(T_RM4_) in IM] + [ln0.49 × mNND(T_RM_ ~ CK) in TC].

The effectiveness of the T_RM_-SIS was evaluated and validated through time-dependent ROC analyses. The AUC values for 1-, 3-, and 5-year DFS were as follows: in the training set, 0.823, 0.794, and 0.742, respectively; in the testing set, 0.687, 0.691, and 0.690, respectively; and in the entire cohort, 0.776, 0.730, and 0.693, respectively. Notably, the T_RM_-SIS system manifested better predictive ability than the TNM staging system (Fig. 3F–H). In survival analyses, NSCLC patients with high T_RM_-SIS presented worse prognosis compared with those with low T_RM_-SIS (mDFS: 33.07 months vs. not reached, *P* < 0.001 in the training set; mDFS: 67.97 months vs. not reached, *P* = 0.048 in the testing set; mDFS: 58.93 months vs. not reached, *P* < 0.001 in the entire cohort, respectively) (Fig. 3I–K; Additional file 1: Table S6). As shown in univariate and multivariate Cox models, T_RM_-SIS [HR = 2.43, 95%CI (1.63–3.60), *P* < 0.001] and tumor stage [stage II vs. stage I: HR = 1.85, 95%CI (1.15–2.98), *P* = 0.011; stage III vs. stage I: HR = 3.10, 95%CI (1.99–4.83), *P* < 0.001] were independent prognostic factors for DFS (Table 1).

**Table 1:** Univariate and multivariate Cox regression analysis of disease-free survival in NSCLC cohort

Variables	Univariate		Multivariate	
	HR (95%CI)	*P* value	HR (95%CI)	*P* value
Age				
≤ 60 years	Ref			
˃60 years	0.87 (0.60, 1.27)	0.469		
Gender				
Male	Ref			
Female	0.78 (0.52, 1.17)	0.224		
Smoking status				
Non- or light smoker	Ref			
Heavy smoker	1.15 (0.77, 1.72)	0.483		
Histological subtype				
LUAD	Ref			
LUSC	1.20 (0.81, 1.78)	0.359		
Tumor stage				
Stage I	Ref			
Stage II	1.63 (1.02, 2.62)	0.043	1.85 (1.15, 2.98)	0.011
Stage III	3.21 (2.06, 5.00)	< 0.001	3.10 (1.99, 4.83)	< 0.001
T_RM_-SIS				
Low	Ref			
High	2.43 (1.64, 3.58)	< 0.001	2.43 (1.63, 3.60)	< 0.001

LUAD, lung adenocarcinoma; LUSC, lung squamous cell carcinoma; T_RM_, tissue-resident memory T cells; SIS, spatial immune signature; HR, hazard ratio; Ref, reference

To improve our ability to stratify patients, we integrated T_RM_-SIS and tumor stage to predict prognosis of patients. The AUC values for 1-, 3-, and 5-year DFS were as follows: in the training set, 0.820, 0.810, and 0.780, respectively; in the testing set, 0.757, 0.703, and 0.711, respectively; and in the entire cohort, 0.770, 0.739, and 0.730, respectively, which indicated better performance (Additional file 1: Figure S3). Collectively, our findings show that spatial features of specific T_RM_ cell subsets may act as a biomarker for the prognosis of patients with NSCLC.

### Validation of the clinical value of CD103 in NSCLC

To verify our results to some extent, we collected 244 eligible cases with stage I-III NSCLC from TCGA database (Additional file 1 Table S7; Additional file 2: Suppl_data2). Similarly, the expression of CD103 was significantly correlated with that of PD-1, Tim-3 and GZMB (Additional file 1: Figure S4A-C). Further, the survival analyses showed that patients in the high CD103 expression group had longer DFS despite no statistical significance (mDFS: not reached vs. not reached, *P* = 0.170) and significantly longer OS (mOS: 65.1 months vs. 31.7 months, *P* = 0.002) compared with those in the low CD103 expression group (Additional file 1: Figure S4D, E).

### Tumor angiogenesis was unfavorable to TRM cells

**Fig. 4:**
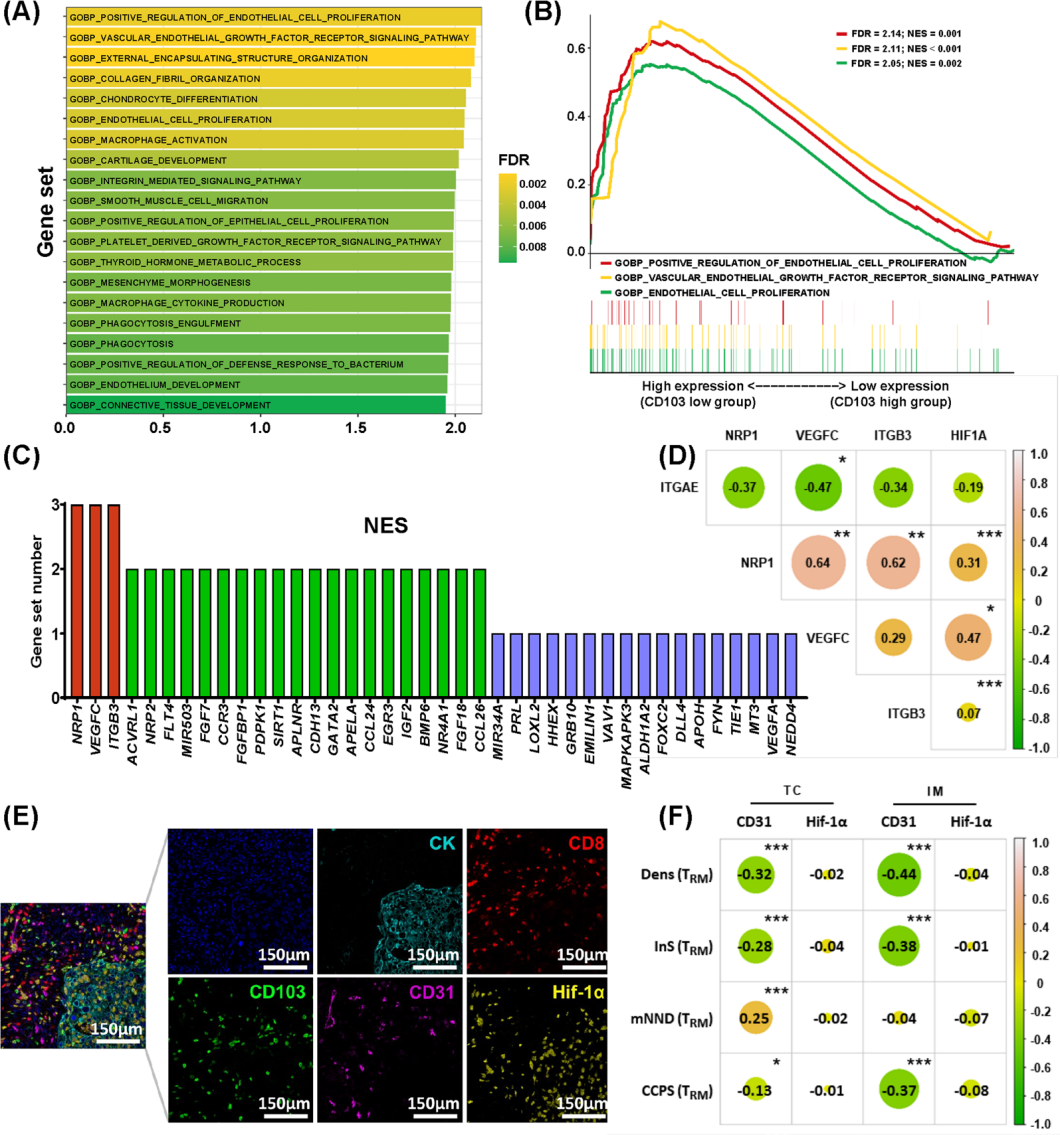
Tumor angiogenesis may impede the infiltration of T_RM_ cells. **A** Gene set map showing the top 20 biological pathways enriched in the low *ITGAE* (coding CD103) expression group in gene set enrichment analysis (GSEA), mostly related to angiogenesis or immunity. **B** Detailed enrichment profiles for the three main angiogenesis-correlated pathways in GSEA. **C** Gene overlapping rate among the main three angiogenesis-correlated pathways in GSEA. **D** Correlation among the expression of CD103, overlapped genes (*NRP1*, *VEGFC*, *ITGB3*), and hypoxia indicator (*HIF1A*). **E** The merged and single-stained images for the panel of multiplex immunofluorescence. **F** Correlation among T_RM_ cells, cancer microvessels (CMVs) (indicated by CD31), and hypoxia (indicated by Hif-1α). The correlation was calculated using the Spearman rank correlation coefficient. *P < 0.05; **P < 0.01; ***P < 0.001

Lung T_RM_ cells have been found to exhibit plasticity in transportation and retention in virus-infected mouse models[32, 33]. For a better understanding of potential regulatory mechanisms and the relevant biological pathways of T_RM_ in the tumor microenvironment, we performed a GSEA using 20 suitable samples with operable NSCLC from the GSE10245 dataset (Additional file 2: Suppl_data3). The 20 tumor samples were divided into high and low CD103 expression groups according to the median normalized count of *ITGAE* (coding CD103 protein). Among a total of 463 gene sets which were significantly enriched between two groups, 371 upregulated gene sets in the low CD103 expression group accounted for the largest proportion (371/463, 80.13%). As shown in the top 20 pathways related to the low expression of CD103, most of them involved the extracellular stromal microenvironment, especially angiogenesis (Fig. 4A). Figure 4B demonstrated the three main angiogenesis-related pathways enriched in the low CD103 expression group: “GOBP_POSITIVE_REGULATION_OF_ENDOTHELIAL_CELL_PRO-LIFERATION” (|NES|= 2.14, FDR q-value = 0.001), “GOBP_VASCULAR_ENDOTHELIAL-_GROWTH_FACTOR_ RECEPTOR_SIGNALING_PATHWAY” (|NES|= 2.11, FDR q-value = 0.001) and “GOBP_ENDO-THELIAL_CELL_PROLIFERATION” (|NES|= 2.05, FDR q-value = 0.002). A close relationship and high overlapping rate between the above three angiogenesis-related pathways enriched in the low CD103 expression group were found (Fig. 4C). Specifically, through leading-edge analysis, a total of 23 genes were found to be significant in at least two pathways (23/40, 57.50%), and *VEGFC*, *NRP1* as well as *ITGB3* overlapped in all three pathways. Furthermore, we conducted a correlation analysis and found that *ITGAE* was negatively correlated with *VEGFC* (*r* = − 0.47, *P* = 0.036), *NRP1* (*r* = − 0.37, *P* = 0.107) and *ITGB3* (*r* = − 0.34, *P* = 0.140) (Fig. 4D). For gene sets positively correlated with CD103, detailed enrichment profiles and overlapped genes among the top three pathways were also depicted (Additional file 1: Figure S5).

Given the results of GSEA, it is rational to assume that tumor angiogenesis impeded T_RM_. Based on this assumption, we performed another panel of mIF staining in the SCHI-NSCLC cohort to investigate the effect of cancer microvessels (CMVs) on the density and spatial architecture of T_RM_ cells (Fig. 4E). CD31 was used as an indicator of CMVs’ endothelium, while Hif-1α as an indicator of cellular hypoxia. In the correlation analyses, we found that the CMVs were negatively correlated with the density of T_RM_ cells (*r* = − 0.32, *P* < 0.001 in TC; *r* = − 0.44, *P* < 0.001 in IM), the InS of T_RM_ cells (*r* = − 0.28, *P* < 0.001 in TC; *r* = − 0.38, *P* < 0.001 in IM) and the CCPS of T_RM_ cells (*r* = − 0.13, *P* = 0.039 in TC; *r* = − 0.37, P < 0.001 in IM) while positively with the mNND from T_RM_ cells to cancer cells (*r* = 0.25, *P* < 0.001 in TC) (Fig. 4F). In contrast, no significant correlation was observed between CMVs and T_non-RM_ cells (Additional file 1: Figure S6). Interestingly, the density and spatial architecture of T_RM_ were not associated with Hif-1α (Fig. 4F). Moreover, compared with the low T_RM_-SIS group, the high T_RM_-SIS group had a significantly higher density of CMVs in IM (*P* = 0.006) (Additional file 1: Figure S7A, B). Survival analysis also showed that high density of CMVs (*P* = 0.054 in TC; *P* = 0.007 in IM) and high level of hypoxia (*P* = 0.018 in TC; *P* = 0.006 in IM) were associated with poorer prognosis in NSCLC patients (Additional file 1: Figure S7C–F).

## Discussion

In this study, using mIF, we deciphered the spatial architecture of CD103^+^CD8^+^ T_RM_ functional subsets in the TME of 274 patients with NSCLC. Our results demonstrated that the T_RM_ was a population of cells with significant heterogeneity in their functional state and spatial distribution.

To the best of our knowledge, this is the first study to reveal the spatial distribution of T_RM_ subsets. Utilizing mIF to detect multiple protein markers simultaneously, we can characterize T_RM_ subsets more precisely than the traditional immunohistochemistry method. More importantly, it can provide unreported spatial information that cannot be revealed through dissociative techniques such as single-cell RNA sequencing or flow cytometry. Our results confirmed the previous findings histologically that CD103 expression was linked to the magnitude of CD8^+^T cell infiltration and cytotoxic T cell responses in human lung cancer [7, 8]. A few studies have demonstrated that CD103^+^T_RM_ cells are more abundant in the epithelial compartment of prostate cancer and CD39^+^T_RM_ cells are closer to cancer cells in melanoma, which provides partial support for our results [34, 35].

As previously mentioned, the role of T_RM_ in tumorigenesis and prognosis remains controversial. However, few studies have focused on the internal heterogeneity of T_RM_ cells. We divided T_RM_ cells into four subsets based on different expression patterns of immune checkpoints (PD-1 and Tim-3) and found a significant heterogeneity among them. Specifically, T_RM2_ had the strongest cytotoxicity but the lowest cell density, while T_RM4_ had the weakest cytotoxicity. T_RM3_ and T_RM4_ had a higher InS, shorter mNND to cancer cells and higher CCPS than T_RM1_ and T_RM2_. More importantly, T_RM4_ was associated with an unfavorable prognosis of NSCLC patients. These results suggested that T_RM4_ may be in a terminally dysfunctional state, T_RM2_ in a pre-dysfunctional state, and T_RM3_ in an intermediate state. The great heterogeneity among T_RM_ functional subsets is one of the reasons for the inconsistent prognostic relevance of T_RM_ cells and the tumor progression after T_RM_ adoptive transfer [7–12]. Based on current understanding of the PD-(L)1 axis as an immune brake, the superior functionality of T_RM2_ was unexpected [36]. Likewise, the transcriptome profiling of human lung cancer also showed that PD-1^+^T_RM_ possessed superior antitumor activity, suggesting potentially unknown mechanisms contributing to activation of T cells [13]. We have to underline that although our results presented a gradient from T_RM1_ to T_RM4_, it does not mean that the four T_RM_ subsets are in a progressive evolutionary relationship. Through temporal single-cell tracing, Liu et al. thought that pre-dysfunctional T cells were unlikely to be derived from terminally dysfunctional cells [37]. In addition, Tim3^+^PD1^+^CD8^+^T cells did not respond well to anti-PD-(L)1 immunotherapy [38, 39]. Unfortunately, as a monotherapy, the Tim-3 blockade did not show substantial clinical benefit. However, in mouse models of melanoma, colorectal cancer and acute myeloid leukemia, combined blocking of Tim-3 and PD-(L)1 pathways restored T-cell response better than blocking PD-(L)1 pathway alone [40]. Several phase I clinical trials combining Tim-3 and PD-(L)1 monoclonal antibody have shown modest antitumor activity in lung cancer (NCT03099109; NCT02608268) [41, 42], and a bispecific antibody targeting both Tim-3 and PD-1 (R07121661) has also entered phase I clinical trials (NCT03708328). Therefore, improving the postoperative outcomes of NSCLC patients with high infiltration of T_RM4_ needs further studies.

**Fig. 5:**
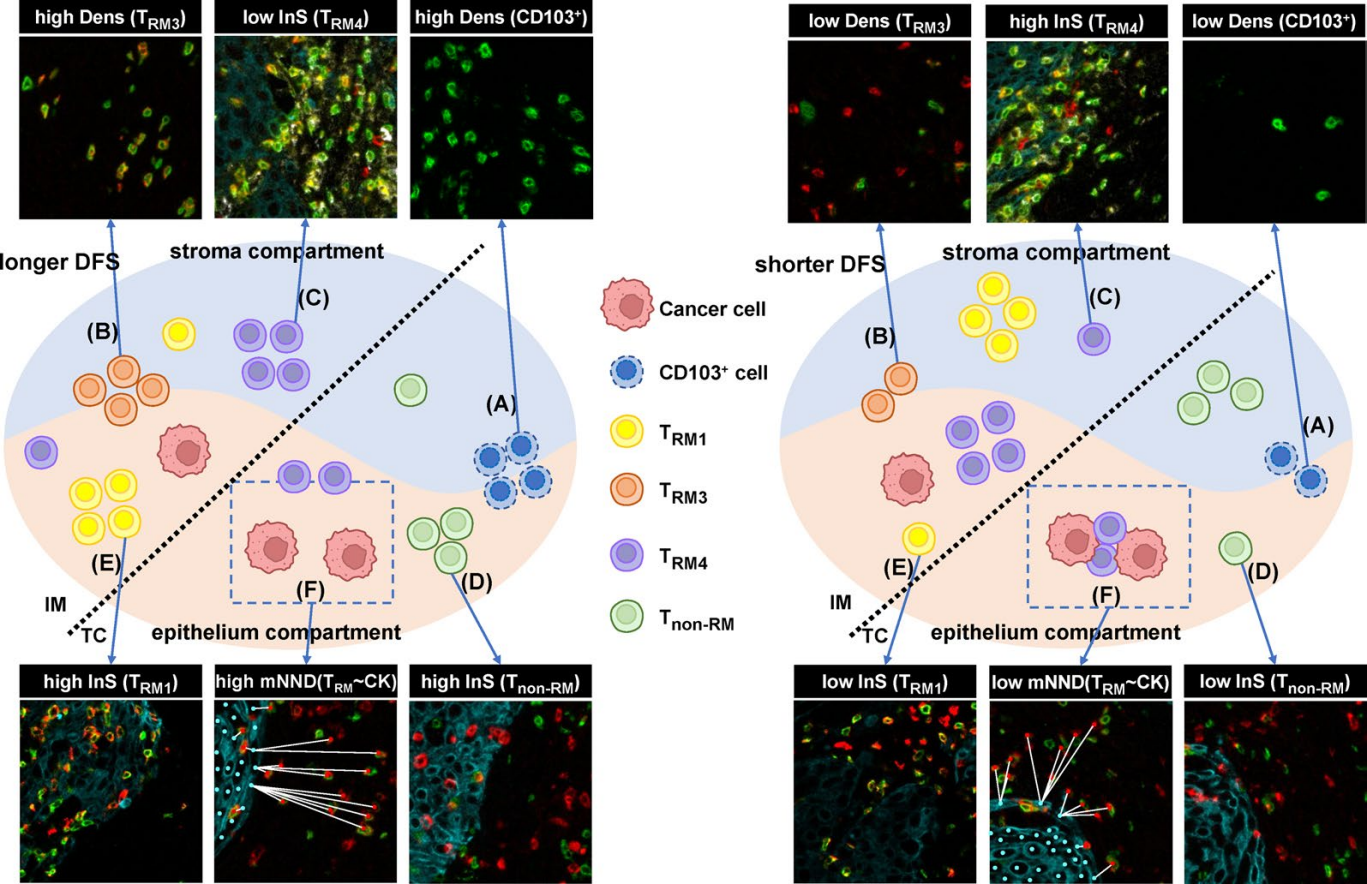
Proposed model of T_RM_-based spatial immune signature predicting DFS of patients with non-small cell lung cancer. Higher density of CD103^+^ cells in TC (**A**), higher density of T_RM3_ cells in IM (**B**), higher infiltration score of T_non-RM_ cells in TC (**C**), higher infiltration score of T_RM1_ cells in IM (**D**), lower infiltration score of T_RM4_ cells in IM (**E**) and higher mean nearest neighboring distance from T_RM_ cells to cancer cells in TC (**F**) are associated with longer disease-free survival (DFS)

In the setting of the most stringent selection criteria to control the overfitting and the overcomplexity of our predictive model, which was to take the intersection of LASSO, XGBoost and RF results, the T_RM_-related features we selected could be more representative [43]. Six features were screened out in all three methods and used to construct a T_RM_-SIS (Fig. 5). Importantly, T_RM_-SIS and tumor stage were independent prognostic factors for DFS. Thus, the model combining T_RM_-SIS and tumor stage showed a better performance in patient stratification in our cohort. A clinical implication behind our model is that perioperative treatment or increased monitoring may be worth considering in patients with high T_RM_-SIS due to their high risk of recurrence. Although patients receiving perioperative treatment were not included in our model, the inclusion of these patients in predictive models could be of importance in further studies.

Considering that the intratumoral T_RM_ cells are removed with surgery, the reason why T_RM_-SIS can affect the long-term prognosis of postoperative patients remains unclear. The potential explanation may be that it acts as a biomarker for the tumor with a “hot” immune environment or identifying patient with a long-term systemic antitumor immunity. Once a tumor is removed after radical surgery, immune surveillance may rebuild and take advantage. Intriguingly, previous studies have found that lung T_RM_ could migrate to the draining lymph nodes and enhance immune responses of the nodes in virus-infected mouse models, suggesting a potential T_RM_ storage pool in draining lymph nodes and a plasticity for T_RM_ cells retention [32, 33]. More efforts are needed to investigate the retention and transportation of intratumoral T_RM_, which will help in the development of new antitumor targets.

Cancer vasculature is a vital pipeline to transport immune cells into malignant tissue [44]. Through GSEA, we found tumor angiogenesis may play a pivotal role in the regulation of T_RM_. Furthermore, utilizing mIF to quantify CMVs and T_RM_, we verified the results of GSEA at the level of cellular pathology, which strengthened the evidence that CMVs may act as an immune barrier [45]. Notably, we found that CMVs were significantly negatively correlated with the infiltration of T_RM_ cells but not with that of T_non-RM_ cells, suggesting that the inhibition of CD8^+^T by CMVs may be selective. Moreover, although our previous study demonstrated a significant positive correlation between Hif-1α and CMVs [17], no significant correlation between Hif-1α and reduced infiltration of T_RM_ cells was observed in this study, suggesting that there might be an angiogenesis-independent pathway for tumor hypoxia on T_RM_ cells. Vignali et al. found that tumor hypoxia drove CD39-dependent T-cell dysfunction to limit antitumor immunity [46]. Given the coexpression of CD39 and CD103 in some T_RM_ [47], it is reasonable to speculate that Hif-1α can promote a part of CD103^+^T_RM_ via an angiogenesis-independent pathway. Of note, the negative association between CMVs and T_RM_ appears to be statistically more significant in IM than in TC, suggesting angiogenesis could be one of the mechanisms for local progression in NSCLC.

CMVs are generally thought abnormal. CMVs have been found to hinder CD8^+^T cell trafficking through the upregulation of apoptotic ligands, like Fas-L, and the downregulation of adhesion molecules, such as E-selectin and VCAM-1 [44, 48]. Importantly, antiangiogenic agents could induce the normalization of CMVs and therefore were considered one of the wonderful partners for immunotherapy. For advanced NSCLC, the ABCP regimen (atezolizumab-bevacizumab-carboplatin-paclitaxel) and its analogous regimen (immunotherapy-antiangiogenic agents-chemotherapy) have conferred benefits in overall survival [49]. Two recent phase 2 clinical trials reported promising benefits and acceptable toxicity of neoadjuvant anti-PD-1 antibody combined with antiangiogenic agent in patients with resectable NSCLC [50, 51]. Considering the high level of CMVs in the high T_RM_-SIS group, antiangiogenic therapy in T_RM_-restricted patients may be a promising option to improve outcomes. Thus, it is crucial to clarify the impact of tumor vascular normalization strategies on T_RM_ for promoting immunotherapy.

Some limitations of this study have to be acknowledged. Firstly, this is a single-center, retrospective study, although we validated some of our results in a public cohort. Secondly, although the expression of CD103 has been observed in the majority of T_RM_ cells, CD103^−^T_RM_ also exist [52]. Hence, identification through our phenotypic markers might underestimate the size of the T_RM_ population. Additionally, the lack of tumor adjacent lung tissue or lung tissue from non-cancer patients as control makes it hard to determine whether the features of T_RM_ cells in this study are tumor-specific [53, 54]. Lastly, after clarifying the significance of the spatial architecture of T_RM_ for the prognosis of NSCLC, its relevance for therapeutic efficacy was not elaborated due to the lack of a suitable cohort, which is exactly the orientation requiring more effort.

In summary, this study highlights the significant heterogeneity of T_RM_ cells in their functional state and spatial distribution, which could be a prognostic biomarker for patients with NSCLC. Tumor angiogenesis may inhibit the infiltration of T_RM_ cells, suggesting that targeting tumor angiogenesis to promote T_RM_ cells may be a potential strategy to improve current immunotherapy.

### Supplementary Information

**Additional file 1: Table S1**. Information of regents used in multiplex immunofluorescence test. **Table S2.** Scheme of cell phenotypes in multiplex immunofluorescence detection. **Table S3. **Clinicopathological characteristics of 274 NSCLC patients enrolled in this study.** Table S4.** The ratio of the 90% signal level to the 10% signal level.** Table S5.** Clinicopathological characteristics of 274 NSCLC patients in the training set and testing **set. Table S6.** Discrepancy of the clinicopathological characteristics between patients in high-risk group and low-risk group.** Table S7.** Clinicopathological characteristics of 244 NSCLC patients in the TCGA validation cohort. **Figure S1.** Evaluating heterogeneity of signal intensity of CD8 (**A**), CD103 (**B**), PD-1 (**C**), Tim-3 (**D**), GZMB (**E**) and CK (**F**), DAPI (**G**) and autofluorescence (**H**) across multiplex immunofluorescence (mIF) images. **Figure S2.** Evaluating crosstalk between spectral channels in eight randomly selected mIF images. **Figure S3.** Predicted probability of the recurrence in resectable NSCLC by integrating T_RM_-SIS and tumor stage. **Figure S4.** Validation of the clinical value of CD103 in the TCGA cohort of NSCLC. A–C Close correlation of *ITGAE* (coding CD103) and *PDCD1* (coding PD-1), *HAVCR2* (coding Tim-3) and *GZMB* (coding GZMB). **Figure S5.** Gene set enrichment analysis (GSEA) positively correlated with CD103. **Figure S6.** Correlation among T_non-RM_ cells, cancer microvessels (CMVs) (indicated by CD31), and hypoxia (indicated by Hif-1α). **Figure S7.** Clinical relevance of cancer microvessels and hypoxia.

**Additional file 2: **Source data for the spatial immune signature construction. **Suppl_data1.** Source data for the spatial immune signature construction. **Suppl_data2.** Source data for the TCGA validation cohort. **Suppl_data3.** GEO accession codes of samples in the GSEA.

## Data Availability

All data and materials are published in the manuscript, supplementary materials on the journal website or available from corresponding author upon reasonable request.
