# Supplementary material for: Spatial features of specific CD103+CD8+ tissue-resident memory T cell subsets define the prognosis in patients with non-small cell lung cancer
Source: J Transl Med. 2024 Jan 5;22:27. doi: 10.1186/s12967-023-04839-4 (PMC10770937; doi:10.1186/s12967-023-04839-4)
Supplement: Supplementary file 1 — Additional file 1: Table S1. Information of regents used in multiplex immunofluorescence test. Table S2. Scheme of cell phenotypes in multiplex immunofluorescence detection. Table S3. Clinicopathological characteristics of 274 NSCLC patients enrolled in this study. Table S4. The ratio of the 90% signal level to the 10% signal level. Table S5. Clinicopathological characteristics of 274 NSCLC patients in the training set and testing set. Table S6. Discrepancy of the clinicopathological characteristics between patients in high-risk group and low-risk group. Table S7. Clinicopathological characteristics of 244 NSCLC patients in the TCGA validation cohort. Figure S1. Evaluating heterogeneity of signal intensity of CD8 (A), CD103 (B), PD-1 (C), Tim-3 (D), GZMB (E) and CK (F), DAPI (G) and autofluorescence (H) across multiplex immunofluorescence (mIF) images. Figure S2. Evaluating crosstalk between spectral channels in eight randomly selected mIF images. Figure S3. Predicted probability of the recurrence in resectable NSCLC by integrating TRM-SIS and tumor stage. Figure S4. Validation of the clinical value of CD103 in the TCGA cohort of NSCLC. A–C Close correlation of ITGAE (coding CD103) and PDCD1 (coding PD-1), HAVCR2 (coding Tim-3) and GZMB (coding GZMB). Figure S5. Gene set enrichment analysis (GSEA) positively correlated with CD103. Figure S6. Correlation among Tnon-RM cells, cancer microvessels (CMVs) (indicated by CD31), and hypoxia (indicated by Hif-1α). Figure S7. Clinical relevance of cancer microvessels and hypoxia. [file 12967_2023_4839_MOESM1_ESM.docx]

# Additional Tables and Figures

### Spatial features of specific CD103^+^CD8^+^ tissue-resident memory T cell subsets defined the prognosis in patients with non-small cell lung cancer

Guanqun Yang^1, 2^, Siqi Cai^1, 2^, Mengyu Hu^2, 3^, Chaozhuo Li^2, 4^, Liying Yang^1, 2^, Wei Zhang^2^, Jujie Sun^5^, Ligang Xing^1, 2^, Xiaorong Sun^1, 6*^

^*^ Corresponding author: Xiaorong Sun; Email: radiomd@163.com; Tel: 86-0531-67626287; Address: Department of Nuclear Medicine, Shandong Cancer Hospital and Institute, No.440, Jiyan Road, Huaiyin District, Jinan 250117, China.

| **Additional file 1: *Table S1****. Information of regents used in multiplex immunofluorescence test* | | | |
| --- | --- | --- | --- |
| Reagent | Provider | Identifier | Concentration |
| Anti-pan-CK antibody | ZSGB-BIO | ZM-0069 | 1:200 |
| Anti-CD8 antibody | Abcam | ab199016 | 1:500 |
| Anti-CD103 antibody | Abcam | ab224202 | 1:200 |
| Anti-GZMB antibody | Abcam | ab255598 | 1:2000 |
| Anti-Tim-3 antibody | Cell Signaling Technology | 45208S | 1:100 |
| Anti-PD-1 antibody | ZSGB-BIO | ZM-0381 | 1:1 |
| Anti-CD31 antibody | Abcam | ab76533 | 1:100 |
| Anti-Hif-1ɑ antibody | Abcam | ab51608 | 1:100 |
| Opal Antibody Block | Akoya biosciences | ARD1001EA | 1:1 |
| Opal Polymer HRP | Akoya biosciences | ARH1001EA | 1:100 |
| Amplification Diluent | Akoya biosciences | FP1498 | 1:1 |
| Spectral DAPI | Akoya biosciences | FP1498 | 1:100 |
| Anti-Fade Fluorescence | Abcam | ab104315 | 1:100 |

| **Additional file 1: *Table S2.*** ***Scheme of cell phenotypes in multiplex immunofluorescence detection.*** | | |
| --- | --- | --- |
| **Cell population** | **Cell subpopulation** | ***Marker*s** |
| Cancer cell | N/A | CK^+^ |
| Tissue-resident memory T cell  (T_RM_) | T_RM_ | CD8+CD103+ |
|  | T_RM1_ | CD8^+^CD103^+^PD-1^-^Tim-3^-^ |
|  | T_RM2_ | CD8^+^CD103^+^PD-1^+^Tim-3^-^ |
|  | T_RM3_ | CD8^+^CD103^+^PD-1^-^Tim-3^+^ |
|  | T_RM4_ | CD8^+^CD103^+^PD-1^+^Tim-3^+^ |
| Cancer microvessel (CMV) | N/A | CD31^+^ |
| Hypoxia indicator | N/A | Hif-1α^+^ |
| N/A, not applicable | | |

| **Additional file 1: *Table S3.* Clinicopathological characteristics of 274 NSCLC patients enrolled in this study** | |
| --- | --- |
| Characteristics | Number, n (%) |
| Age, years, n (%) |  |
| ≤ 60 | 123 (44.8) |
| ˃ 60 | 151 (55.1) |
| median (IQR) | 62 (57, 67) |
| Gender, n (%) |  |
| Male | 178 (65.0) |
| Female | 96 (35.0) |
| Smoking status, n (%) |  |
| Non or mild-smoker | 157 (56.6) |
| Heavy-smoker | 117 (43.4) |
| Histological type, n (%) |  |
| LUSC | 95 (34.6) |
| LUAD | 179 (65.3) |
| AJCC Stage, n (%) |  |
| IA | 72 (26.2) |
| IB | 80 (29.1) |
| IIA | 18 (6.5) |
| IIB | 50 (18.2) |
| IIIA | 50 (18.2) |
| IIIB | 4 (1.4) |
| *LUSC, lung squamous cell carcinoma; LUAD, lung adenocarcinoma; IQR, interquartile range.* | |

| Additional file 1: Table S4. The ratio of the 90% signal level to the 10% signal level. | | | | | | | | |
| --- | --- | --- | --- | --- | --- | --- | --- | --- |
| Source | Auto | CD103 | CD8 | CK | DAPI | GZMB | PD-1 | Tim-3 |
| 03_Core[1,1,B] | 12.0 | Inf | Inf | Inf | 49.8 | Inf | Inf | Inf |
| 03_Core[1,5,E] | 12.0 | Inf | Inf | Inf | 51.2 | Inf | Inf | Inf |
| 04_Core[1,2,F] | 12.2 | Inf | Inf | Inf | 55.9 | Inf | Inf | Inf |
| 04_Core[1,7,B] | 13.2 | Inf | Inf | Inf | 57.9 | Inf | Inf | Inf |
| 06_Core[1,2,G] | 15.2 | Inf | Inf | Inf | 61.7 | Inf | Inf | Inf |
| 06_Core[1,10,C] | 19.0 | Inf | Inf | Inf | 59.9 | Inf | Inf | Inf |
| 09_Core[1,4,D] | 16.3 | Inf | Inf | Inf | 57.5 | Inf | Inf | Inf |
| 09_Core[1,8,C] | 13.1 | Inf | Inf | Inf | 48.9 | Inf | Inf | Inf |
| *Note: For Opal fluorophores, a general guideline is that ratios below 30 are considered poor signal-to-noise and need likely be improved.* | | | | | | | | |

| **Additional file 1: Table S5. Clinicopathological characteristics of 274 NSCLC patients in the training set and testing set** | | | |
| --- | --- | --- | --- |
| **Characteristics** | **Training set, n (%)**  **(N = 192)** | **Testing set, n (%)**  **(N = 82)** | ***P* value**  **(Chi-square test)** |
| Age, years, n (%) |  |  | 0.202 |
| ≤ 60 | 91 (47.3) | 32 (39.0) |  |
| ˃ 60 | 101 (52.6) | 50 (60.9) |  |
| median (IQR) |  |  |  |
| Gender, n (%) |  |  | 0.450 |
| Male | 122 (63.5) | 56 (68.2) |  |
| Female | 70 (36.4) | 26 (31.7) |  |
| Smoking status, n (%) |  |  | 0.284 |
| Non or mild-smoker | 106 (55.2) | 51 (62.2) |  |
| Heavy-smoker | 86 (44.8) | 31 (37.8) |  |
| ECOG score, n (%) |  |  | 0.940 |
| 0-1 | 139 (72.3) | 59 (71.9) |  |
| ˃ 1 | 53 (27.6) | 23 (28.0) |  |
| Histological type, n (%) |  |  | 0.875 |
| LUSC | 66 (35.3) | 29 (35.3) |  |
| LUAD | 126 (65.6) | 53 (64.6) |  |
| T stage, n (%) |  |  | 0.901 |
| T1 | 59 (30.7) | 29 (35.3) |  |
| T2 | 112 (58.3) | 45 (54.8) |  |
| T3 | 11 (5.7) | 4 (4.8) |  |
| T4 | 10 (5.2) | 4 (4.8) |  |
| N stage, n (%) |  |  | 0.017 |
| N0 | 125 (65.1) | 64 (78.0) |  |
| N1 | 35 (18.2) | 13 (15.8) |  |
| N2 | 32 (16.6) | 5 (6.0) |  |
| AJCC Stage, n (%) |  |  | 0.096 |
| I | 100 (52.0) | 52 (63.4) |  |
| II | 48 (25.0) | 20 (24.3) |  |
| III | 44 (22.9) | 10 (12.1) |  |
| *LUSC, lung squamous cell carcinoma; LUAD, lung adenocarcinoma; IQR, interquartile range.* | | | |

| **Additional file 1: Table S6. Discrepancy of the clinicopathological characteristics between patients in high-risk group and low-risk group** | | | |
| --- | --- | --- | --- |
| **Characteristics** | **High-risk group, n (%)**  **(N = 127)** | **Low-risk group, n (%)**  **(N = 147)** | ***P* value**  **(Chi-square test)** |
| Age, years, n (%) |  |  | 0.625 |
| ≤ 60 | 55 (43.3) | 68 (46.2) |  |
| ˃ 60 | 72 (56.6) | 79 (53.7) |  |
| Gender, n (%) |  |  | 0.164 |
| Male | 88 (69.2) | 90 (61.2) |  |
| Female | 39 (30.7) | 57 (38.7) |  |
| Smoking status, n (%) |  |  | 0.244 |
| Non or mild-smoker | 68 (53.5) | 89 (60.5) |  |
| Heavy-smoker | 59 (46.5) | 58 (39.5) |  |
| ECOG score, n (%) |  |  | 0.119 |
| 0-1 | 86 (67.7) | 112 (76.1) |  |
| ˃ 1 | 41 (32.2) | 35 (23.8) |  |
| Histological type, n (%) |  |  | 0.313 |
| LUSC | 48 (62.2) | 47 (31.9) |  |
| LUAD | 79 (37.7) | 100 (68.0) |  |
| AJCC Stage, n (%) |  |  | 0.198 |
| I | 100 (53.5) | 84 (57.1) |  |
| II | 48 (20.4) | 42 (28.5) |  |
| III | 44 (25.9) | 21 (14.2) |  |
| T_RM_-SIS |  |  |  |
| Median (IQR) | -1.09 (-1.13, -0.70) | -1.78 (-2.15, -1.65) | ˂ 0.001 |
| *LUSC, lung squamous cell carcinoma; LUAD, lung adenocarcinoma; IQR, interquartile range; T_RM_, tissue-resident memory T cell; T_RM_-SIS, T_RM_-based spatial immune signature.* | | | |

| **Additional file 1: Table S7. Clinicopathological** **characteristics of 244 NSCLC patients in the TCGA validation cohort** | |
| --- | --- |
| Characteristics | Number, n (%) |
| Age, years, n (%) |  |
| ≤ 60 | 61 (25.0) |
| ˃ 60 | 183 (75.0) |
| median (IQR) | 68.5 (60.8, 73.0) |
| Gender, n (%) |  |
| Male | 141 (57.8) |
| Female | 103 (42.2) |
| Histological type, n (%) |  |
| LUSC | 115 (47.1) |
| LUAD | 129 (52.8) |
| T stage, n (%) |  |
| T1 | 66 (27.0) |
| T2 | 133 (54.5) |
| T3 | 36 (14.7) |
| T4 | 9 (3.6) |
| N stage, n (%) |  |
| N0 | 158 (64.8) |
| N1 | 54 (22.1) |
| N2 | 27 (11.0) |
| N3 | 5 (2.0) |
| AJCC Stage, n (%) |  |
| I | 122 (50.0) |
| II | 74 (30.3) |
| III | 48 (19.7) |
| *LUSC, lung squamous cell carcinoma; LUAD, lung adenocarcinoma; IQR, interquartile range.* | |


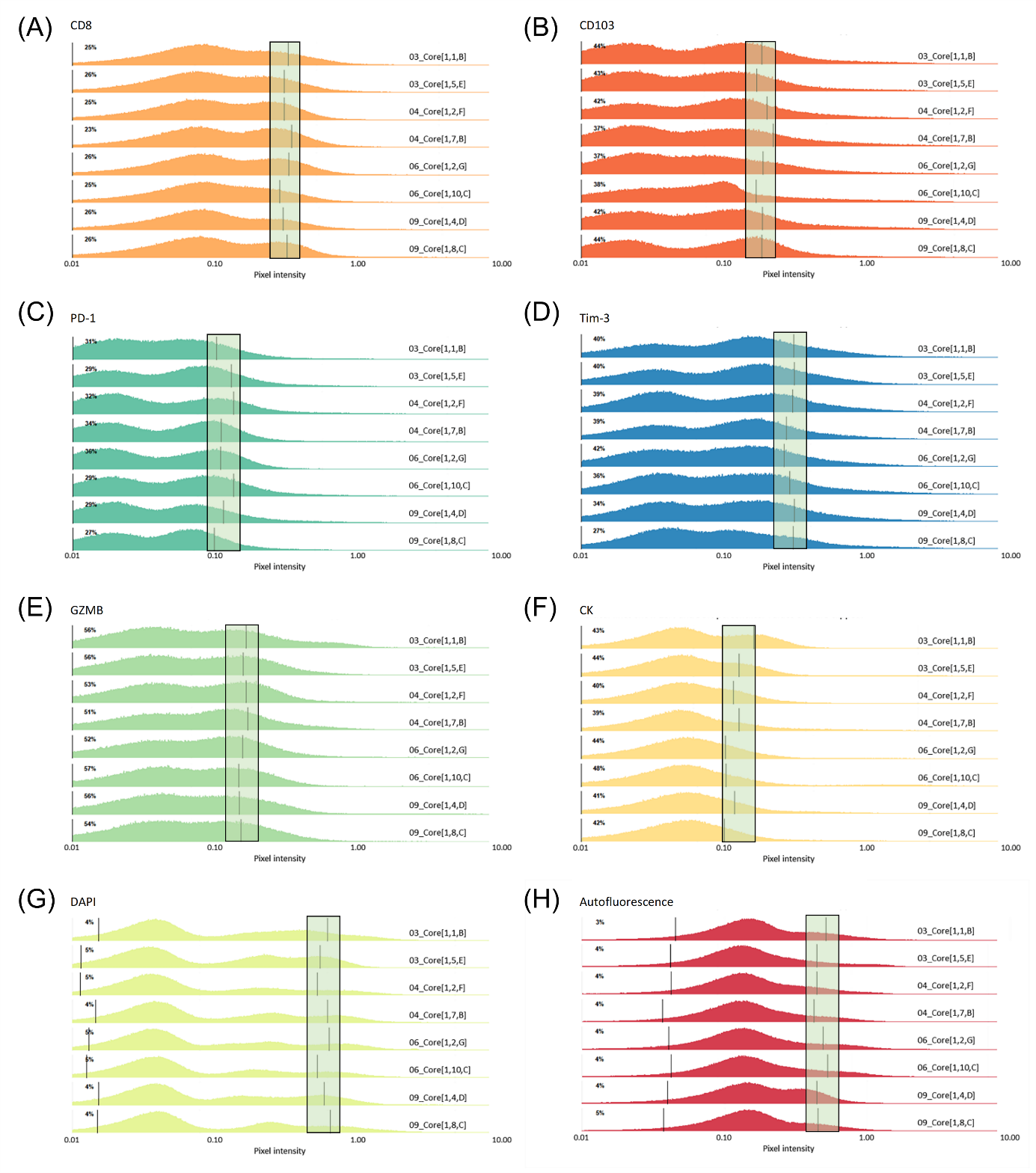


**Additional file 1: Figure S1.** Evaluating heterogeneity of signal intensity of CD8 (A), CD103 (B), PD-1 (C), Tim-3 (D), GZMB (E) and CK (F), DAPI (G) and autofluorescence (H) across multiplex immunofluorescence (mIF) images. The top 99.9% percentile pixel is usually reflective of a ‘positive’ signal. Vertical lines show 10% and 99.9% percentiles. Eight mIF multispectral images in random four tissue microarrays were randomly selected for evaluation and it was demonstrated that there was no outlier or large-scale difference in staining intensity across them.


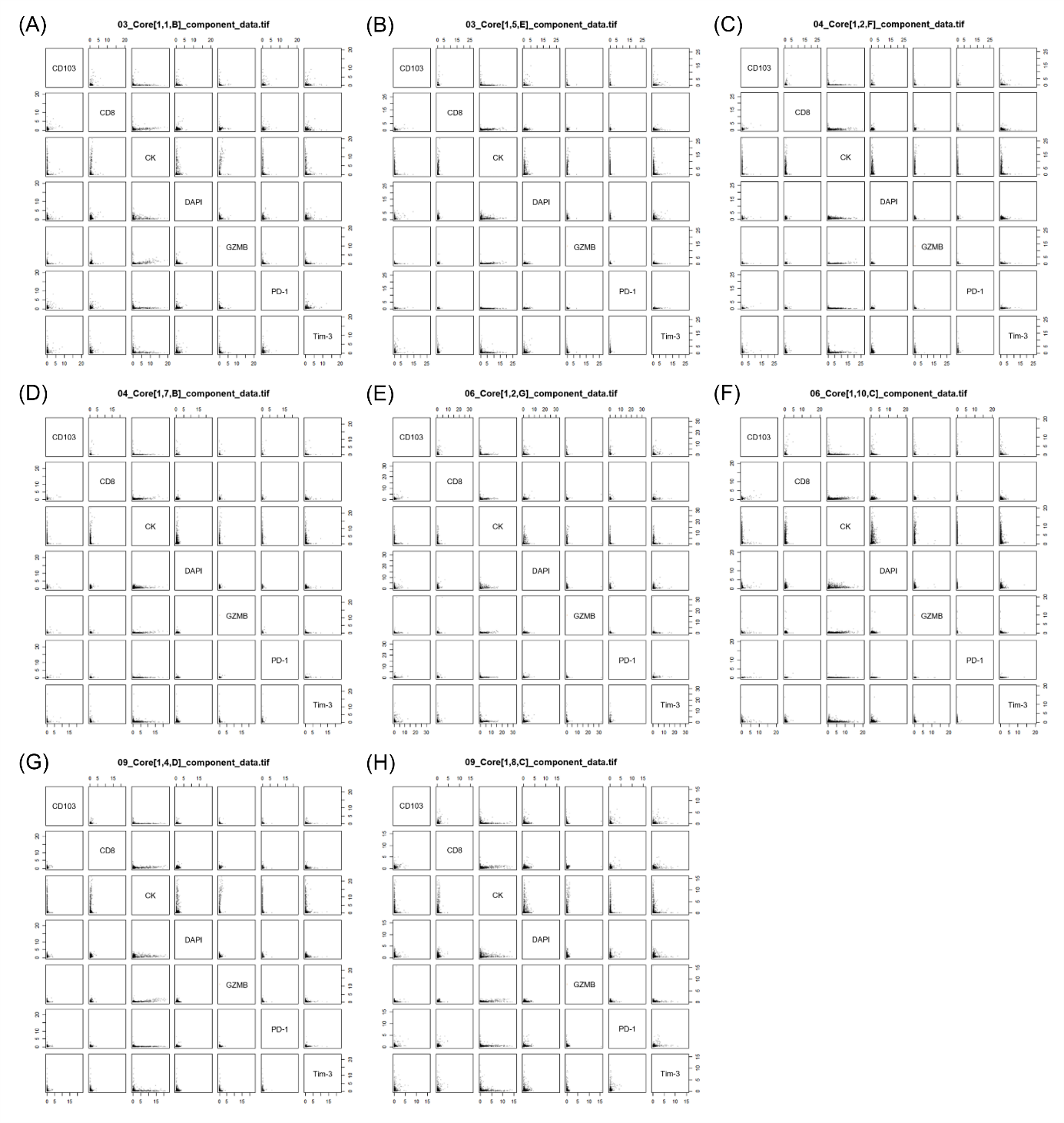


**Additional file 1: Figure S2.** Evaluating crosstalk between spectral channels in eight randomly selected mIF images. This figure can give a quick indication of whether one spectral signal is bleeding into another channel after unmixing. Dots along the horizontal or vertical axis (i.e. little to no correlation) will indicate clean unmixing, while dots along the diagonal (i.e. tight correlation) indicate crosstalk between channels. Eight mIF multispectral images in random four tissue microarrays were randomly selected for evaluation and it was demonstrated that all spectral channels were well unmixed from each other.


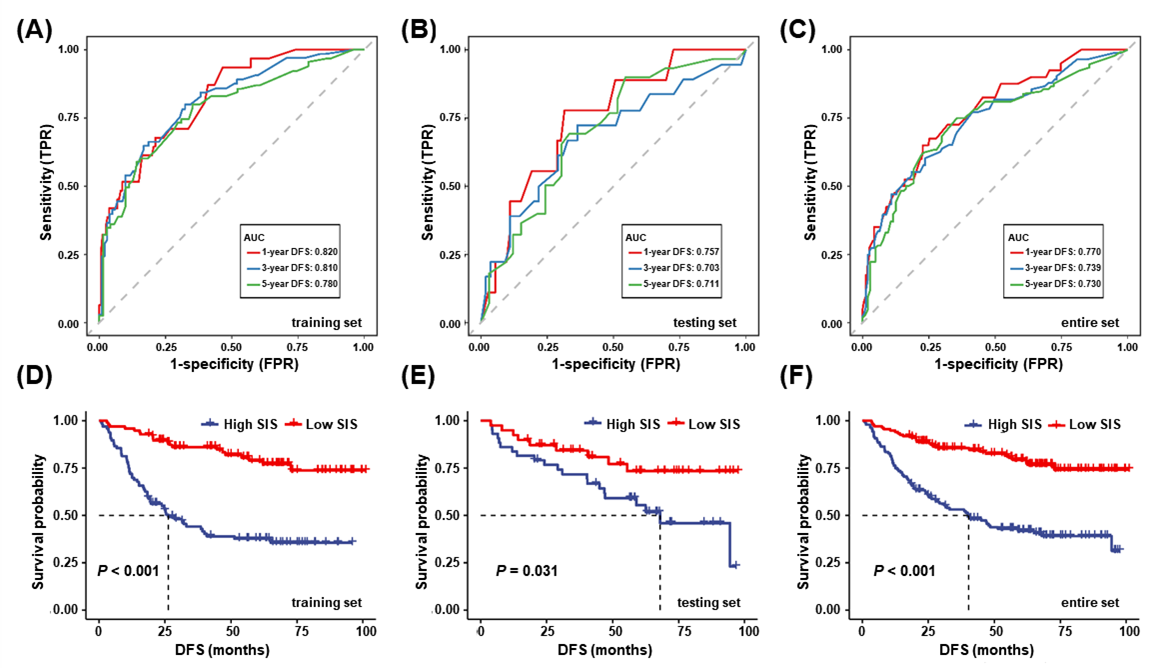


**Additional file 1: Figure S3. Predicted probability of the recurrence in resectable NSCLC by integrating T_RM_-SIS and tumor stage.** (A-C) Time-dependent ROC curves and AUC values for prediction of recurrence risk at 1, 3 and 5 years in the training set, testing set and entire cohort. The AUC values for 1-, 3-, and 5-year DFS were 0.820, 0.810, and 0.780, respectively, in the training set; 0.757, 0.703, and 0.711, respectively, in the testing set; and 0.770, 0.739, and 0.730, respectively, in the entire cohort. (D-F) Survival analyses in the training set, testing set and entire cohort. Statistical significance was calculated using the log-rank test. AUC, area under the curve; DFS, disease-free survival; ROC, receiver operating characteristic.


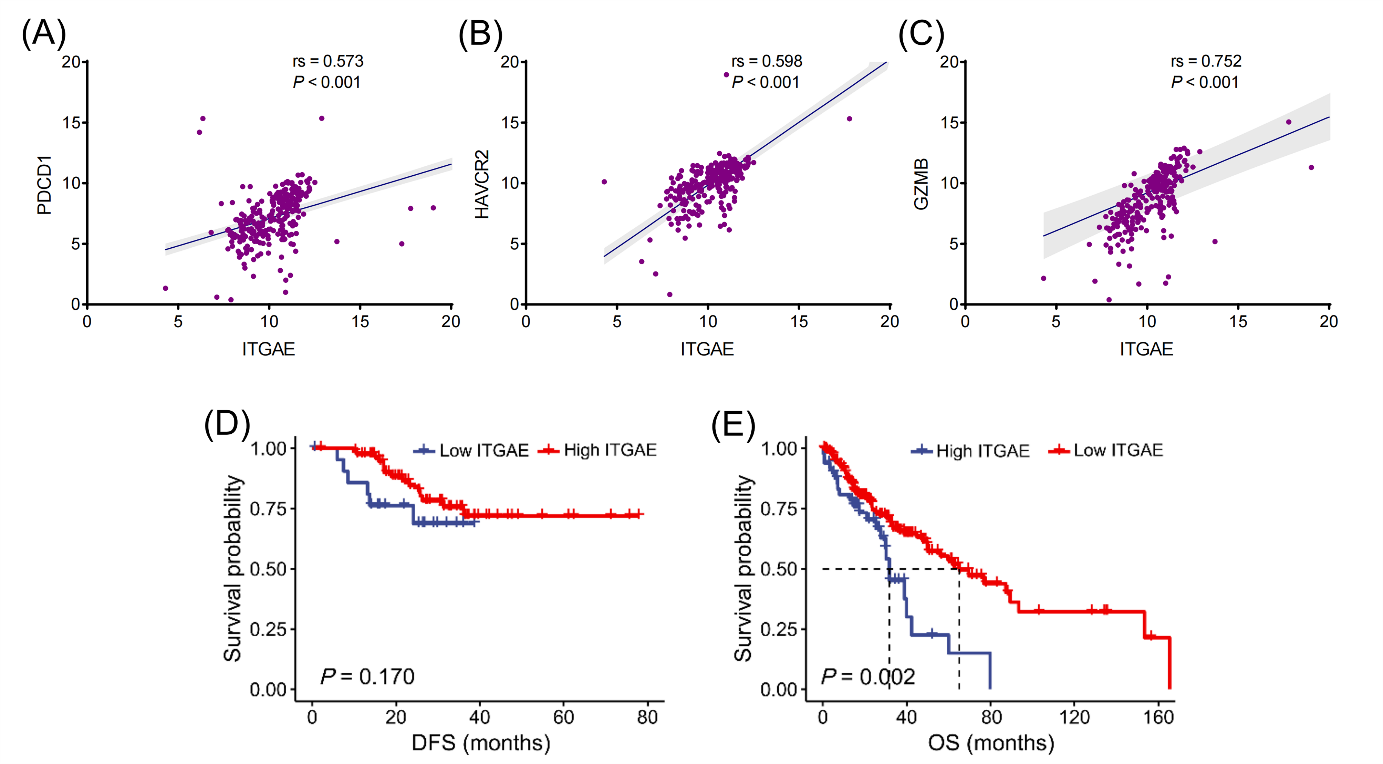


**Additional file 1: Figure S4.** Validation of the clinical value of CD103 in the TCGA cohort of NSCLC. (A-C) Close correlation of *ITGAE* (coding CD103) and *PDCD1* (coding PD-1), *HAVCR2* (coding Tim-3) and *GZMB* (coding GZMB). The correlation was calculated using the Spearman rank correlation coefficient. (D-E) Survival analyses and log-rank tests based on the expression of CD103 for DFS and OS. Statistical significance was calculated using the log-rank test. DFS, disease-free survival; OS, overall survival.


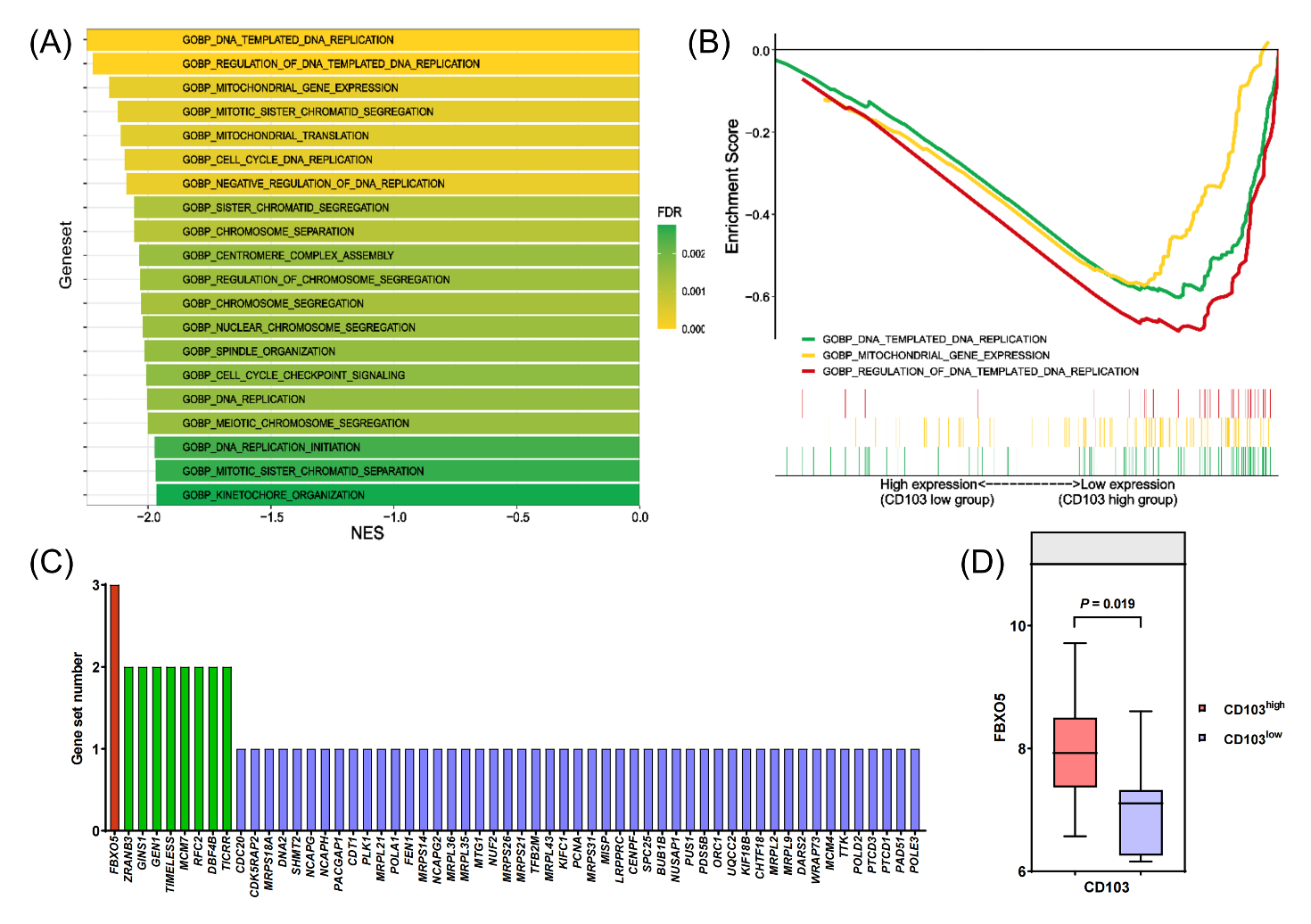


**Additional file 1: Figure S5.** Gene set enrichment analysis (GSEA) positively correlated with CD103. (A) Gene set map showing the Top 20 biological pathways enriched in the high CD103 (coded by *ITGAE*) expression group. (B) Detailed enrichment profiles for the Top 3 pathways enriched in the high CD103 expression group. (C) Gene overlapping rate among the Top 3 pathways in GSEA. (D) Different expression between the high CD103 expression group and low CD103 expression group. Statistical significance was calculated using the Mann-Whitney U test.


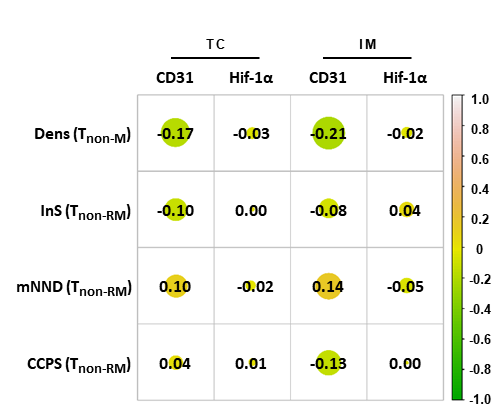


**Additional file 1: Figure S6.** Correlation among T_non-RM_ cells, cancer microvessels (CMVs) (indicated by CD31), and hypoxia (indicated by Hif-1α). Heat map showing no significant correlation between T_non-RM_ cells with CMVs and Hif-1α. The correlation was calculated using the Spearman rank correlation coefficient.


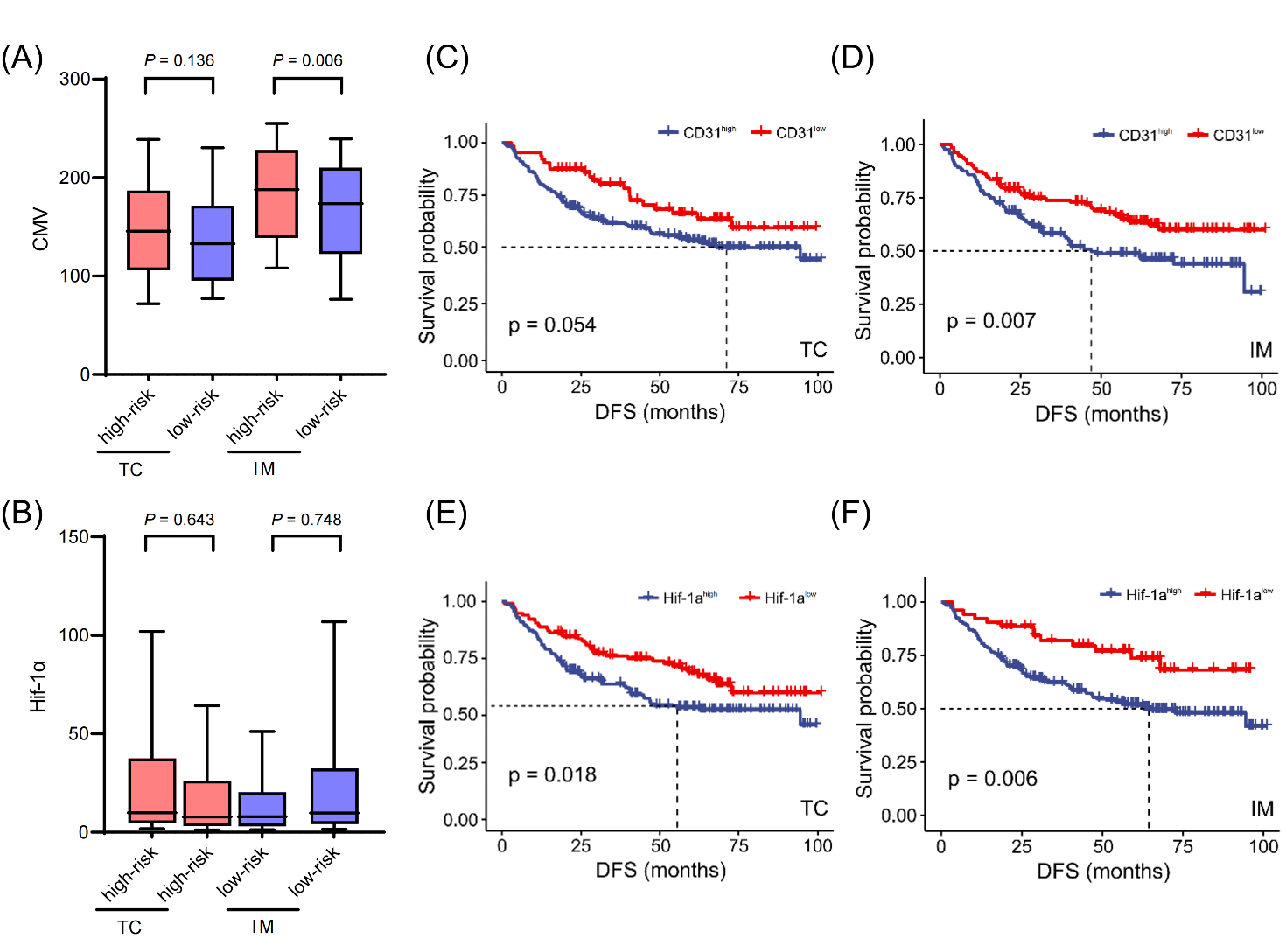


**Additional file 1: Figure S7.** Clinical relevance of cancer microvessels and hypoxia. (A-B) Difference of CD31 and Hif-1α between the high-risk group and low-risk group defined by the T_RM_-SIS in this study. Statistical significance was calculated using the Mann-Whitney U test. (C-F) Survival analyses based on CMVs in TC, CMVs in IM, Hif-1α in TC and Hif-1α in IM for DFS. Statistical significance was calculated using the log-rank test. TC, tumor center; IM, invasive margin; DFS, disease-free survival.
